# Correction: The population genetics of human disease: The case of recessive, lethal mutations

**DOI:** 10.1371/journal.pgen.1007499

**Published:** 2018-07-02

**Authors:** Carlos Eduardo G. Amorim, Ziyue Gao, Zachary Baker, José Francisco Diesel, Yuval B. Simons, Imran S. Haque, Joseph Pickrell, Molly Przeworski

The authors note that the citation of Wright (1937) and Nei (1968) is incorrect in the “Models of mutation-selection balance” section in Results. Wright (1937) not only showed the equilibrium frequency of a deleterious allele for a population of infinite size (Eq 1) but also derived the full equilibrium frequency distribution of a deleterious allele under a finite effective population size. He also derived the mean (Eq 2) allele frequency of a fully recessive deleterious allele and gave approximations for large and small effective population sizes. Nei (1968) further derived the approximate variance (Eq 3) in allele frequency of a fully recessive mutation by using Wright’s general formula. The authors confirm that this error does not affect the conclusions of the article.
