# Supplementary material for: The population genetics of human disease: The case of recessive, lethal mutations
Source: PLoS Genet. 2017 Sep 28;13(9):e1006915. doi: 10.1371/journal.pgen.1006915 (PMC5619689; doi:10.1371/journal.pgen.1006915)
Supplement: S1 Table — (DOCX) [file pgen.1006915.s001.docx]

**Table S1. List of lethal, recessive Mendelian diseases considered in this study.**

| **Gene** | **Disease** | **Phenotype OMIM number** | **Number of variants that passed “variant filter”^a^** |
| --- | --- | --- | --- |
| *ALS2* | Amyotrophic lateral sclerosis 2, juvenile | 205100 | 0 |
| *ASPA* | Canavan disease | 271900 | 11 |
| *ASS1* | Citrullinemia, classic | 215700 | 7 |
| *BCS1L* | Gracile syndrome | 603358 | 1 |
| *CFTR* | Cystic fibrosis | 219700 | 135 |
| *CLN5* | Ceroid lipofuscinosis, neuronal, 5 | 256731 | 11 |
| *DHCR7* | Smith-Lemli-Opitz syndrome | 270400 | 17 |
| *ERCC8* | Cockayne syndrome A | 216400 | 3 |
| *FAH* | Tyrosinemia, type I | 276700 | 12 |
| *FKTN* | Muscular dystrophy-dystroglycanopathy (congenital with brain and eye anomalies), type A, 4 | 253800 | 1 |
| *GAA* | Glycogen storage disease II | 232300 | 21 |
| *GALC* | Krabbe disease | 245200 | 9 |
| *GAN* | Giant axonal neuropathy 1, autosomal recessive | 256850 | 4 |
| *GBA* | Gaucher disease | 608013 | 0 |
| *GBE1* | Glycogen storage disease IV | 232500 | 6 |
| *GNPTAB* | Mucolipidosis II | 252500 | 0 |
| *HEXA* | Tay-Sachs disease | 272800 | 13 |
| *HSD17B4* | D-bifunctional protein deficiency | 261515 | 3 |
| *IDUA* | Hurler-Scheie syndrome | 607015 | 12 |
|  | Hurler syndrome | 607014 |  |
| *LAMA3* | Epidermolysis bullosa, junctional, Herlitz type | 226700 | 1 |
| *LAMB3* | Epidermolysis bullosa, junctional, Herlitz type | 226700 | 8 |
| *LAMC2* | Epidermolysis bullosa, junctional, Herlitz type | 226700 | 0 |
| *LARGE* | Muscular dystrophy-dystroglycanopathy (congenital with brain and eye anomalies), type A, 6 | 613154 | 0 |
| *MCOLN1* | Mucolipidosis IV | 252650 | 0 |
| *NPC1* | Niemann-pick disease, type C1 | 257220 | 24 |
| *NPHP3* | Nephronophthisis 3 | 604387 | 0 |
| *NPHP4* | Nephronophthisis 4 | 606966 | 0 |
| *OSTM1* | Osteopetrosis, autosomal recessive 5 | 259720 | 0 |
| *PEX7* | Rhizomelic chondrodysplasia punctata, type 1 | 215100 | 7 |
| *PLA2G6* | Neurodegeneration with brain iron accumulation 2A | 256600 | 3 |
| *POLG* | Mitochondrial dna depletion syndrome 4A (Alpers type) | 203700 | 5 |
| *POMGNT1* | Muscular dystrophy-dystroglycanopathy (congenital with brain and eye anomalies), type A, 3 | 253280 | 12 |
| *POMT1* | Muscular dystrophy-dystroglycanopathy (congenital with brain and eye anomalies), type A, 1 | 236670 | 0 |
| *POMT2* | Muscular dystrophy-dystroglycanopathy (congenital with brain and eye anomalies), type A, 2 | 613150 | 0 |
| *PPT1* | Ceroid lipofuscinosis, neuronal, 1 | 256730 | 19 |
| *PRF1* | Hemophagocytic lymphohistiocytosis, familial, 2 | 603553 | 8 |
| *SLC22A5* | Carnitine deficiency, systemic primary | 212140 | 18 |
| *SMARCAL1* | Schimke immunoosseous dysplasia | 242900 | 4 |
| *SMPD1* | Niemann-Pick disease, type A | 257200 | 5 |
| *STAR* | Lipoid congenital adrenal hyperplasia | 201710 | 9 |
| *TCIRG1* | Osteopetrosis, autosomal recessive 1 | 259700 | 1 |
| *TK2* | Mitochondrial DNA depletion syndrome 2 (myopathic type) | 609560 | 22 |
| *TNFSF11* | Osteopetrosis, autosomal recessive 2 | 259710 | 0 |
| *TPP1* | Ceroid lipofuscinosis, neuronal, 2 | 204500 | 5 |
| *ZMPSTE24* | Restrictive dermopathy, lethal | 275210 | 0 |

^a^This refers to the number of variants in each gene that were associated with the severe form of the corresponding disease, with an early onset reported and no indication of incomplete penetrance or effect in heterozygote carriers (see Methods for details).
