# Supplementary material for: The population genetics of human disease: The case of recessive, lethal mutations
Source: PLoS Genet. 2017 Sep 28;13(9):e1006915. doi: 10.1371/journal.pgen.1006915 (PMC5619689; doi:10.1371/journal.pgen.1006915)
Supplement: S4 Table — (DOCX) [file pgen.1006915.s004.docx]

**Table S4. P-values for each gene estimated by simulation, under a model of mutation-selection balance with a plausible demographic history.**

| **Gene** | **P-value** |
| --- | --- |
| *ASPA* | 0.052 |
| *ASS1* | 0.272 |
| *CFTR* | 0.001 |
| *CLN5* | 0.770 |
| *DHCR7* | 0.056 |
| *ERCC8* | 0.054 |
| *FAH* | 0.040 |
| *GAA* | 0.802 |
| *GALC* | 0.560 |
| *GAN* | 1 |
| *GBE1* | 0.302 |
| *HEXA* | 1 |
| *HSD17B4* | 0.096 |
| *IDUA* | 0.008 |
| *LAMB3* | 0.032 |
| *NPC1* | 0.340 |
| *PEX7* | 0.134 |
| *POLG* | 0.010 |
| *POMGNT1* | 1 |
| *PPT1* | 0.046 |
| *PRF1* | 0.244 |
| *SLC22A5* | 0.924 |
| *SMARCAL1* | 0.286 |
| *SMPD1* | 0.016 |
| *STAR* | 0.906 |
| *TK2* | 0.584 |
| *TPP1* | 0.752 |
