# Supplementary material for: The population genetics of human disease: The case of recessive, lethal mutations
Source: PLoS Genet. 2017 Sep 28;13(9):e1006915. doi: 10.1371/journal.pgen.1006915 (PMC5619689; doi:10.1371/journal.pgen.1006915)
Supplement: S5 Table — (DOCX) [file pgen.1006915.s005.docx]

**Table S5.** **Phenotypic effect of mouse knock-outs (see main text)**

| **Gene** | **Human disease** | **OMIM number** | **Phenotype of affected human cases^a^** | **Phenotype of homozygous knockout mice^b^** | **Phenotype of heterozygous knockout mice^b^** |
| --- | --- | --- | --- | --- | --- |
| *ASS1* | Citrullinemia | 215700 | Very high concentration of the amino-acid citrulline in serum, spinal fluid, and urine. | Complete neonatal lethality, abnormal circulating amino-acid level, increased circulating ammonia level. | Abnormal circulating amino-acid level. |
| *CFTR* | Cystic fibrosis | 219700 | Disruption of exocrine function of the pancreas, intestinal glands (meconium ileus), biliary tree (biliary cirrhosis), bronchial glands (chronic bronchopulmonary infection with emphysema) and sweat glands (high sweat electrolyte with depletion in a hot environment). Infertility occurs in males and females. | Partial postnatal lethality, aphagia, pancreatic acinar cell atrophy, abnormal intestine morphology, abnormal digestive system physiology, abnormal gland morphology, acute pancreas inflammation, weight loss, distended abdomen, abnormal ion homeostasis, enlarged gallbladder, abnormal respiratory system physiology, lacrimal gland atrophy. | Impaired fertilization, decreased litter size. |
| *DHCR7* | Smith-Lemli-Opitz syndrome | 270400 | Multiple congenital malformation and mental retardation syndrome. | Complete neonatal lethality, abnormal suckling behavior, weakness, abnormal nasal cavity morphology, fetal growth retardation, cyanosis, abnormal brain development, distended urinary bladder. | Abnormal cholesterol level, syndactyly, partial embryonic lethality, decreased brain size. |
| *NPC1* | Niemann-Pick disease, type C1 | 257220 | Lipid storage disorder characterized by progressive neurodegeneration. | Premature death, abnormal Purkinje cell morphology, increased brain cholesterol level, increased liver cholesterol level, abnormal macrophage morphology, abnormal microglial cell activation, abnormal lipid homeostasis, decreased body weight, impaired coordination. | Increased brain cholesterol level. |
| *POLG* | Alpers syndrome | 203700 | Clinical triad of psychomotor retardation, intractable epilepsy, and liver failure in infants and young children. Pathologic findings include neuronal loss in the cerebral gray matter with reactive astrocytosis and liver cirrhosis. | Premature death, abnormal mitochondrial physiology, decreased thymocyte number, abnormal lymphopoiesis, macrocytic anemia, abnormal erythroid lineage cell morphology. | Abnormal bone marrow cell physiology, increased B cell derived lymphoma incidence. |
| *PRF1* | Hemophagocytic lymphohistiocytosis | 603553 | Immune dysregulation characterized clinically by fever, edema, hepatosplenomegaly, and liver dysfunction. Neurologic impairment, seizures, and ataxia are frequent. | Increased activated T cell number, decreased cytotoxic T cell cytolysis, abnormal cytokine secretion, decreased susceptibility to autoimmune diabetes, increased susceptibility to viral infection, premature death, complete postnatal lethality, liver inflammation, CNS inflammation, abnormal circulating cytokine level, decreased leukocyte cell number. | Insulitis, periinsulitis, impaired natural killer cell mediated cytotoxicity. |
| *SLC22A5* | Carnitine deficiency | 212140 | This results in impaired fatty acid oxidation in skeletal and heart muscle. In addition, renal wasting of carnitine results in low serum levels and diminished hepatic uptake of carnitine by passive diffusion, which impairs ketogenesis. | Premature death, enlarged liver, hepatic steatosis, increased triglyceride level, decreased circulating carnitine level, impaired lipolysis, decreased body weight, enlarged heart. | Decreased circulating carnitine level, impaired lipolysis. |
| *SMPD1* | Niemann-Pick disease, type A | 257200 | The clinical phenotype for type A ranges from a severe infantile form with neurologic degeneration resulting in death usually by 3 years of age. | Premature death, ataxia, lethargy, abnormal apoptosis, decreased body weight, increased macrophage derived foam cell number, abnormal lipid homeostasis, increased susceptibility to bacterial infection, decreased brain size. | Abnormal immune system cell morphology, abnormal neuron differentiation, abnormal depression-related behavior. |

Phenotypes obtained from [1]^a^ and [2]^b^

1. Online Mendelian Inheritance in Man O (2016). Baltimore, MD: McKusick-Nathans Institute of Genetic Medicine, Johns Hopkins University.

2. Eppig JT, Blake JA, Bult CJ, Kadin JA, Richardson JE (2015) The Mouse Genome Database (MGD): facilitating mouse as a model for human biology and disease. Nucleic Acids Res 43: D726-736.
