# Supplementary figures and images for: The population genetics of human disease: The case of recessive, lethal mutations
Source: PLoS Genet. 2017 Sep 28;13(9):e1006915. doi: 10.1371/journal.pgen.1006915 (PMC5619689; doi:10.1371/journal.pgen.1006915)

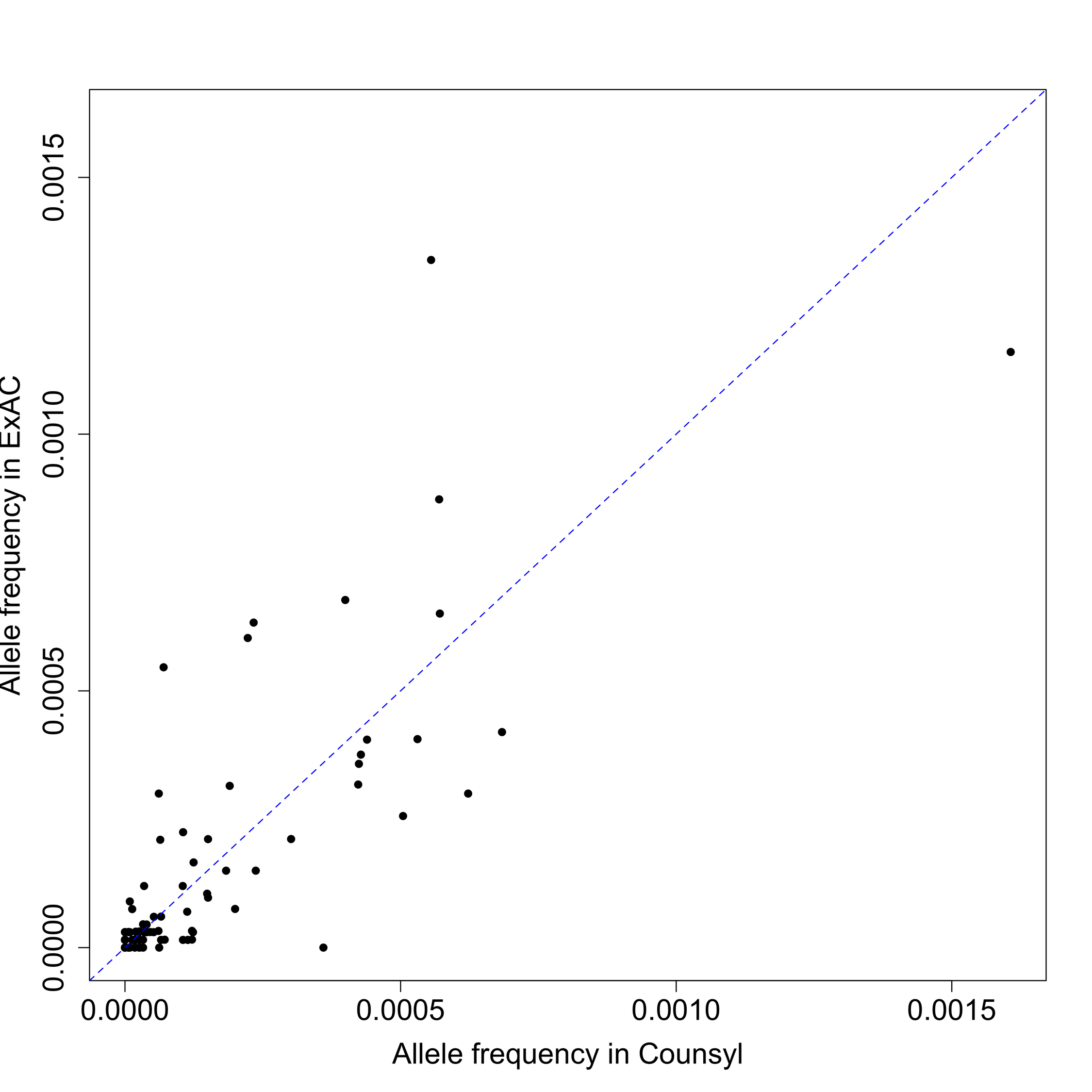

Supplement: S1 Fig — Shown are the allele frequencies for 91 variants associated with lethal, recessive diseases, as estimated from 33,370 individuals of non-Finnish, European ancestry in the Exome Aggregation Consortium (ExAC) database [23] and 76,314 European-ancestry individuals from a genetic testing laboratory (Counsyl [20]) (see Methods). Points lie on the dashed blue line if the allele frequencies in Counsyl and ExAC are the same. (TIF) [file pgen.1006915.s006.tif]

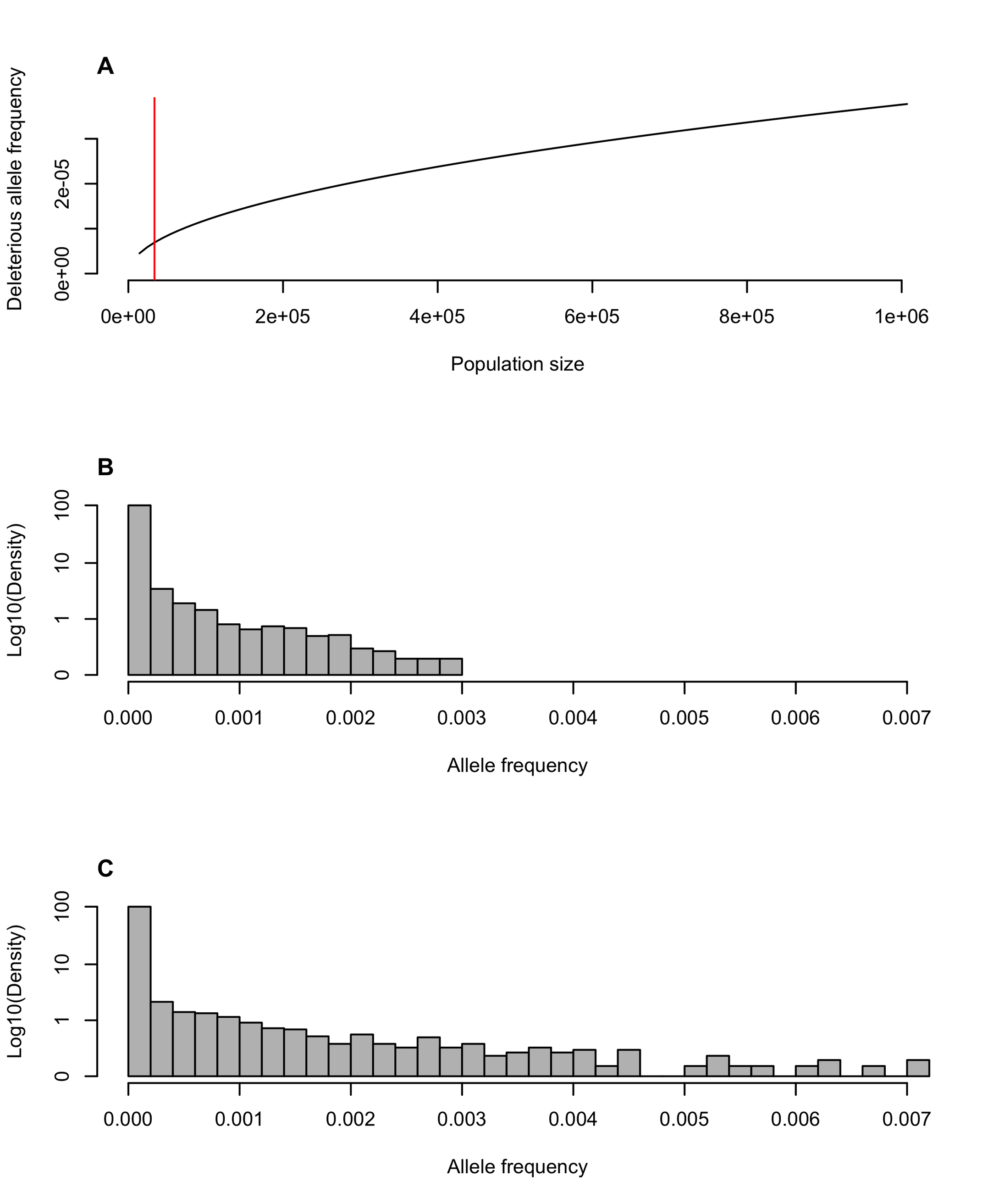

Supplement: S2 Fig — (A) Population mean allele frequency as a function of effective population size, under a model of constant population size. The X-axis range corresponds to the range of effective population size over time estimated in [25]. The red bar indicates the value of a constant population size at which the mean allele frequency is the same as in simulations, for an average mutation rate of 1.5 x10^-8^ per bp per generation [34]. (B-C) The allele frequency distribution (in grey) is presented for 2 x 10^6^ simulations based on (B) the complex demographic scenario inferred by Tennessen et al. [25] for the evolution of European populations based on simulations (see Methods) and of (C) the finite, constant size population model, with N set to 35,651 individuals to match the mean allele frequency with (B). Both models assume complete lethality (s = 1) and recessivity (h = 0). (TIF) [file pgen.1006915.s007.tif]

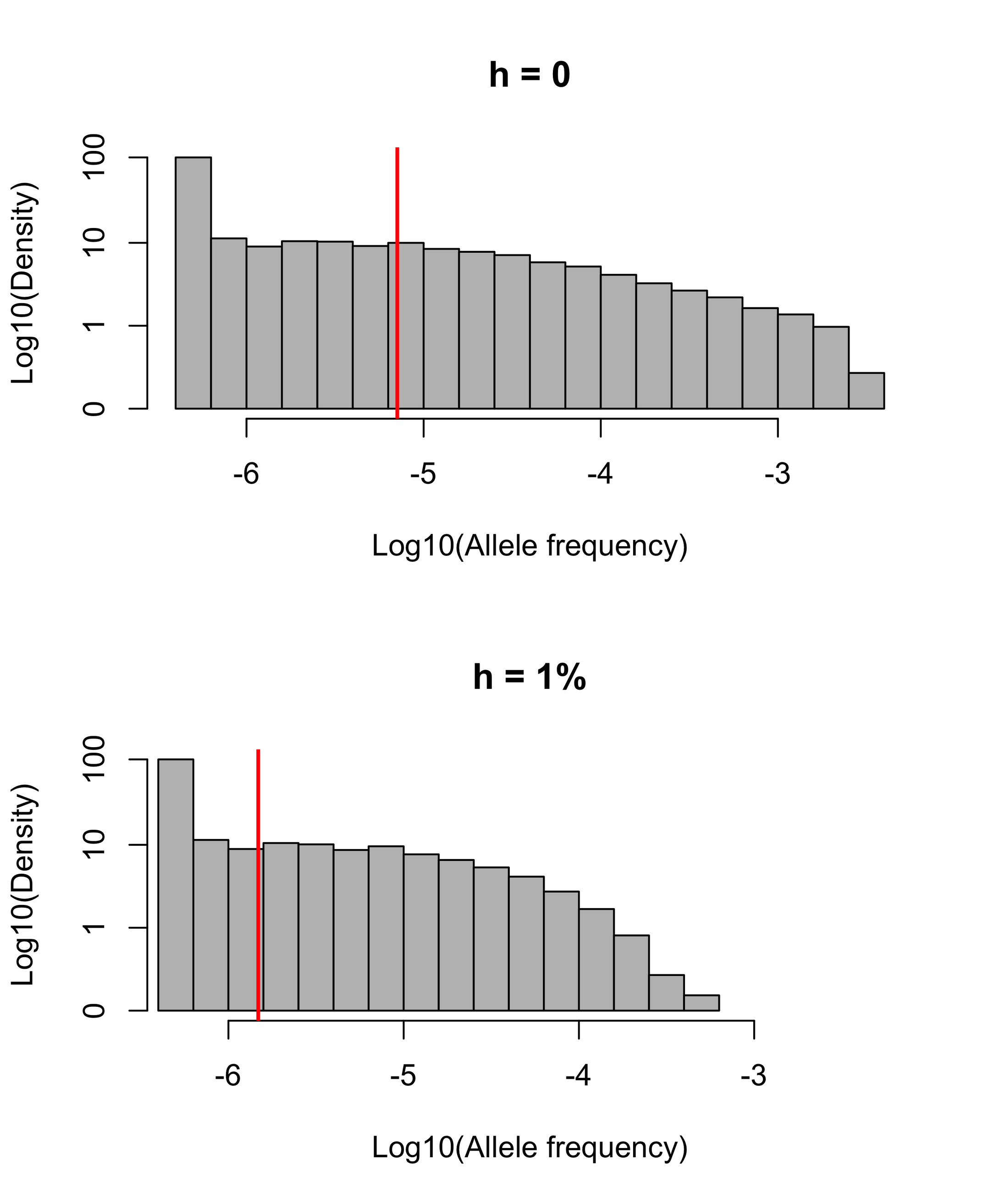

Supplement: S3 Fig — Shown in each case is the distribution of the deleterious allele frequencies in the population, generated from 100,000 simulations. Means are represented by red vertical bars. For visualization, an allele frequency of q = 0 is set to 0.5 x 10^−6^. When a small fitness effect in heterozygotes is considered in the simulations, the mean allele frequency decreases by 79% relative to no effect. The two distributions differ significantly by a Kolmogorov-Smirnov test (p-value < 10−15). The mutation rate u was set to 1.5 x 10^−8^ per bp per generation, reflective of the mean mutation rate for exons [34]. (TIF) [file pgen.1006915.s008.tif]

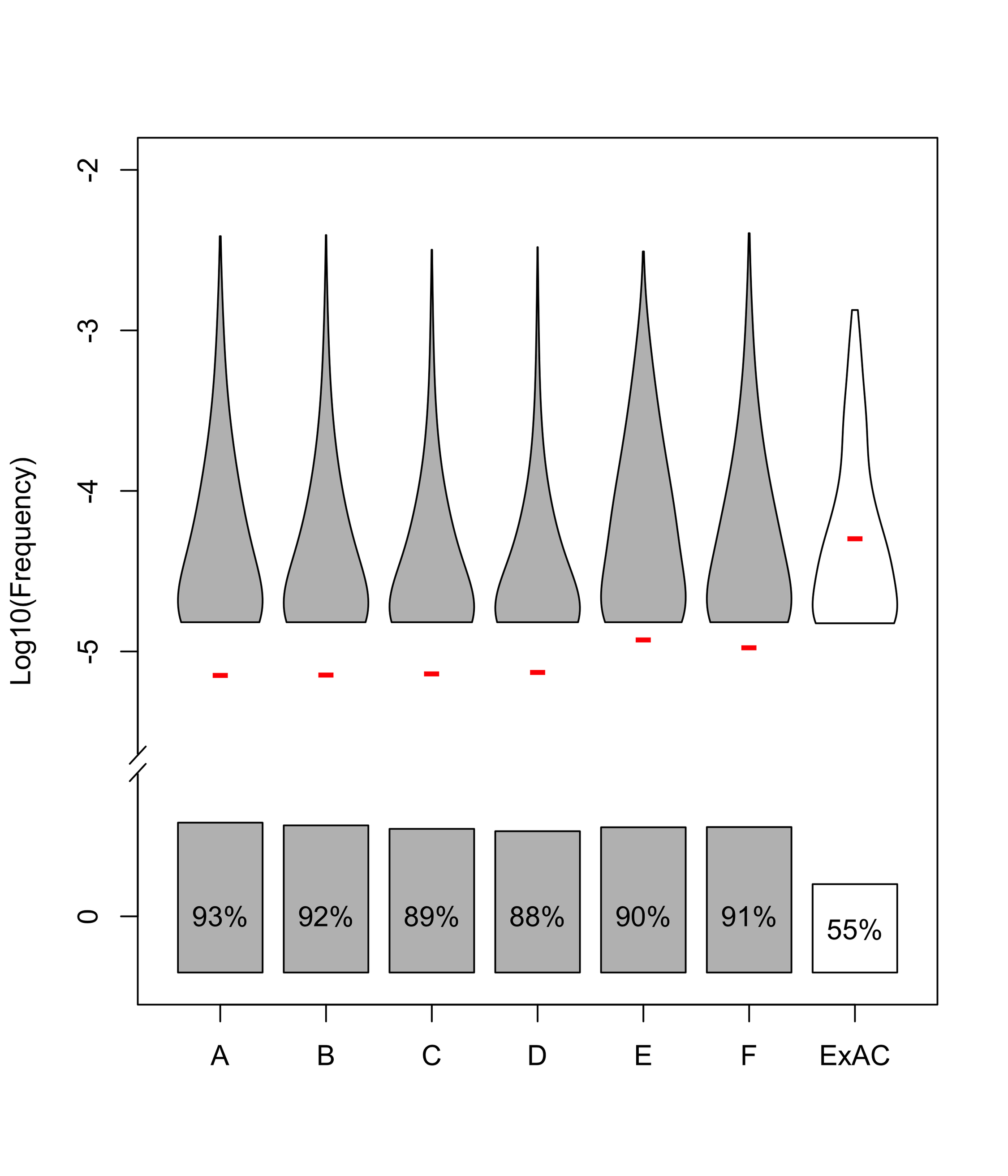

Supplement: S4 Fig — Tennessen et al. [25] inferred the present effective population size of Europeans to be 512,000 individuals based on a mutation rate of 2.36 x 10^−8^ per bp per generation. We rescaled the parameters of this model based on a lower mutation rate estimate of 1.2 x 10^−8^ [18] and show the expected distribution of sample allele frequencies of recessive, lethal mutations in column A. We further considered the effect of larger population sizes at present (2-, 4-, and 10-fold increase, denoted by columns B, C and D respectively), keeping other rescaled demographic parameters the same as in A. We also included a model (E) where rapid growth begins immediately after the out-of-Africa bottleneck, representing a more extreme scenario of population growth in comparison to the two-stage and more gradual scenario proposed by Tennessen et al. (2012). For A-E, we drew the mutation rate M from a lognormal distribution with parameters set as in Eq 8, with u = 1.5 x 10^-8^ (as implemented for Fig 2; see Methods). Model F considers a larger u (2.25 x 10^−8^, i.e., a 1.5-fold increase from A-E), with all other parameters (e.g., variance in mutation rates across simulations, the demographic model) the same as in column A. The observed sample allele frequency distribution of 385 disease mutations in ExAC is shown in white. Violin plots show the density distribution of the log10 allele frequencies for variants that were segregating in these samples, whereas boxes indicate the proportion of sites for which the deleterious mutation was not observed segregating in the sample. All distributions differ significantly from one another (i.e., all p-values are < 10−15 by a Kolmogorov-Smirnov test). (TIF) [file pgen.1006915.s009.tif]

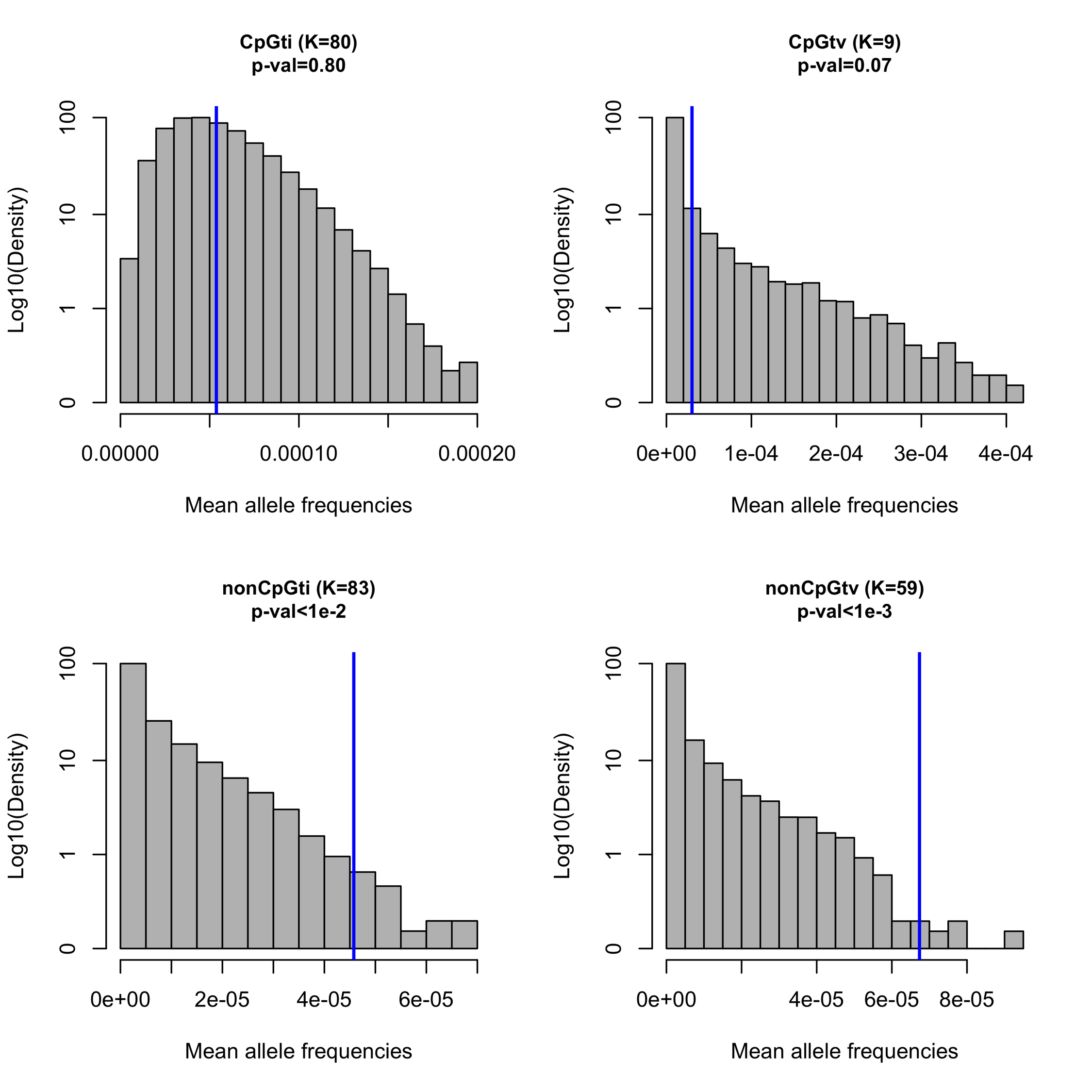

Supplement: S5 Fig — As in Fig 2, the four panels correspond to four different mutation types. The title of the panel indicates the mutation type, followed by K, the total number of mutations of that type, with p-values for the difference between observed and expected mean frequencies below. Distributions in grey are for 100,000 observations of the expected mean sample allele frequencies across K mutations, and were obtained from simulations based on a plausible demographic model for European populations [25] (see Methods). Blue bars represent the observed values estimated from 33,370 individuals of European ancestry from ExAC. As opposed to in Fig 2, here, we did not include mutations present in two genes (CFTR and DHCR7) that were outliers in the gene-level analysis (Fig 3) and were reported elsewhere to be carried by healthy homozygous individuals [24]. (TIF) [file pgen.1006915.s010.tif]

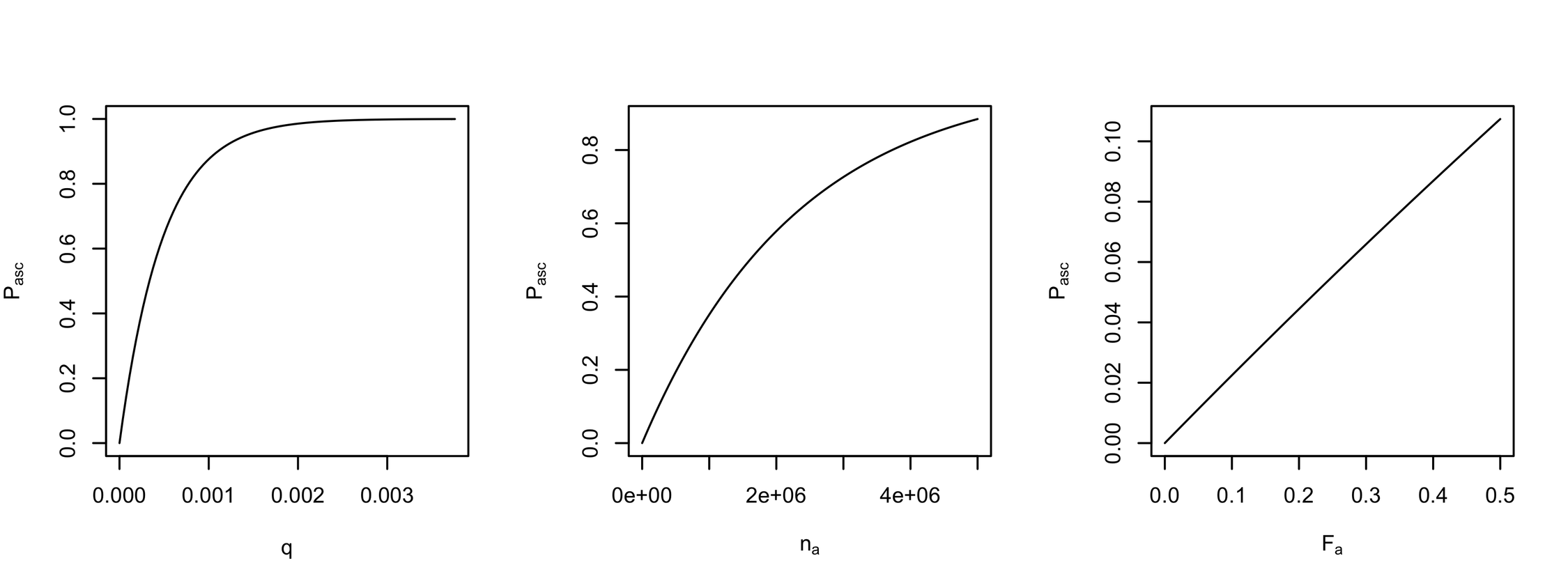

Supplement: S6 Fig — In each case, we let only one parameter (q, na or Fa) vary, while fixing the others at q = 7.10 x 10^−6^ (corresponding to the mean allele frequency from simulations), na = 10,000, and Fa = 1/16 (corresponding to marriage between first cousins, a plausible scenario for a population with widespread inbreeding). (TIF) [file pgen.1006915.s011.tif]

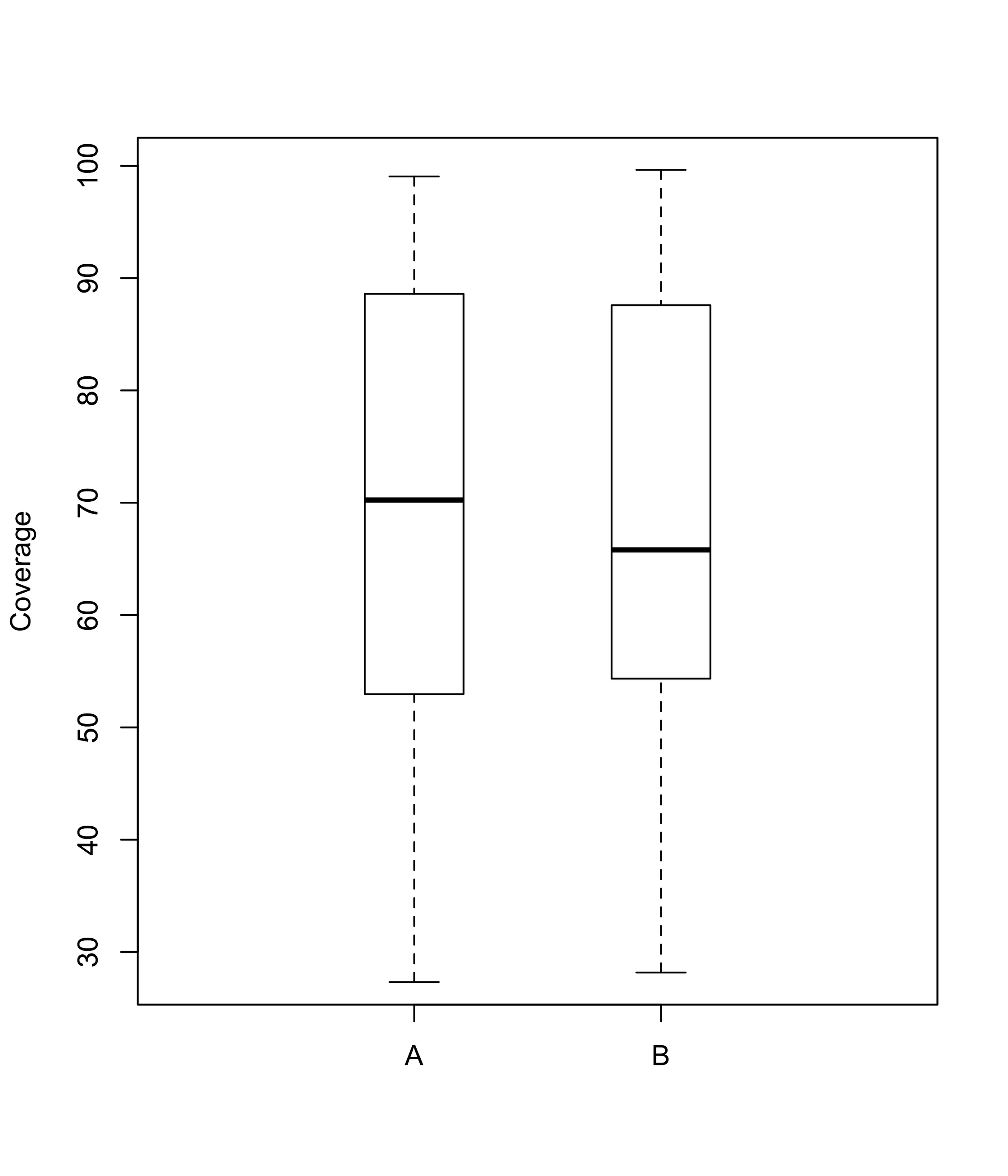

Supplement: S7 Fig — Box plots show the mean (black bar) and the lower and upper quartiles for (A) the 248 sites with non-zero sample frequencies in ExAC, for which the number of sequenced non-Finnish European individuals was reported (n = 32,881) and (B) the 137 sites for which we did not have this information. Since distributions of depth of coverage are similar between the two sets (by a Kolmogorov–Smirnov test, p-value = 0.90), we assumed that 32,881 individuals were sequenced at all sites, and used this number to subsample simulations to match the sample size of the ExAC data. (TIF) [file pgen.1006915.s012.tif]
